# Exsanguinating upper GI bleeds due to Unusual Arteriovenous Malformation (AVM) of stomach and spleen: a case report

**DOI:** 10.1186/1749-7922-4-15

**Published:** 2009-05-01

**Authors:** Mohammad Iqbal Khan, Muhammad Tariq Baqai, Mohammad Fahd Baqai, Naveed Mufti

**Affiliations:** 1 Department of surgery, Islamic International Medical College, Rawalpindi, Pakistan; 2 Department of medicine, Islamic International Medical College, Rawalpindi, Pakistan; 3 Department of medicine, Pakistan Institute of Medical Sciences, Islamabad, Pakistan; 4 Department of Obs & Gynae, Pakistan Institute of Medical Sciences, Islamabad, Pakistan

## Abstract

**Background:**

In this paper we are reporting one case of exsanguinating upper gastrointestinal tract (GIT) bleed requiring massive blood transfusion and immediate life saving surgery.

**Case presentation:**

*A *30 years old female, 12 weeks pregnant was referred to our hospital from the earth-quake affected area of Kashmir with history of upper abdominal pain, haematemesis and melaena for one week. After stabilizing the patient, upper gastro-intestinal endoscopy was performed. It revealed gastric ulcer just distal to the gastro-esophageal junction on the lesser curvature. Biopsy from the ulcer edge led to profuse spurting of the blood and patient went into state of shock. Immediate resuscitation led to rebleeding and recurrence of post haemorrahagic shock.

**Conclusion:**

The patient was immediately explored and total gastrectectomy with splenectomy concluded as life saving procedure. A review of literature was conducted to make this report possible.

## Introduction

Gastrointestinal bleeding is a commonly encountered emergency. Common causes include bleeding peptic ulcers, gastric erosions and esophageal varices. Rare causes include arteriovenous malformation (AVM) of the gastrointestinal tract. With increasing availability of endoscopy and elective angiography AVM is being more frequently recognized. Literature search shows since 1884 about 42 cases have been reported so far worldwide. Upper GI bleeding caused by AVM usually presents as massive haematemesis or chronic iron deficiency anaemia. Non-specific endoscopic appearances make the diagnosis difficult. Therapeutic embolisation offers a better chance of stopping hemorrhage. However, in emergency situations, surgeon may be forced to perform life saving exploration and procedures if selective angiography is not available or unhelpful and when patient with AVM causing massive haemorrhage required surgical arrest of bleeding.

## Case report

A 30 years old lady with 12 weeks gestational amenorrhea was referred to our hospital with history of upper abdominal pain, haematemesis and melaena for last one week. After stabilization upper gastro- intestinal endoscopy was performed. It revealed lesion resembling gastric ulcer on the lesser curvature just distal to gastro- oesophageal junction. Biopsy from the edge of the lesion led to profuse spurting of the blood from the site and the patient went into shock. Resuscitation was done but haemodynamic instability persisted. Immediate exploration was done by mid-line abdominal incision which revealed grossly distended tense stomach. Gastrotomy led to evacuation of 3 to 4 liter of blood. Multiple spurts of blood on posterior wall about 5 cm. from the gastro-oesophageal junction were observed. Under running of these spurts aggravated the haemorrhage. Stomach was packed and mobilized, revealing multiple dilated sub-serosal vessels along the posterior and inferior wall extending from Gastro-oesophagial junction to pylorus. Hilum of the spleen also showed multiple dilated vessels which also bled during the mobilization of the stomach. Total gastrectomy and splenectomy with Roux-NY oesophagojejunostomy was performed. Fourteen units of blood and twelve units of fresh frozen plasma were transfused during the pere operative period.

## Histopathology

**Figure 1:**
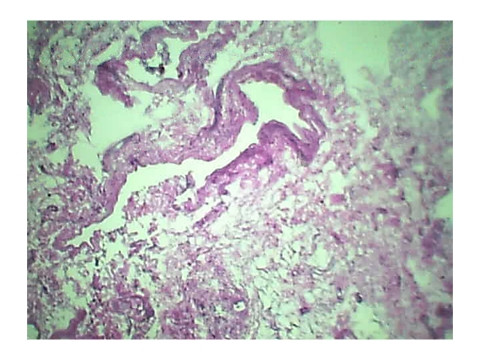
**Histopathology of Stomach highlights overlying gastric mucosa displaying reactive changes**.

**Figure 2:**
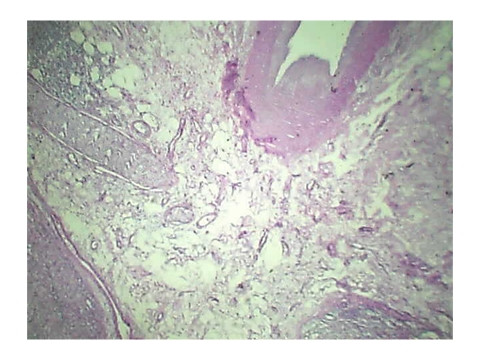
**Histopathology of Stomach highlights overlying gastric mucosa displaying reactive changes**.

**Figure 3:**
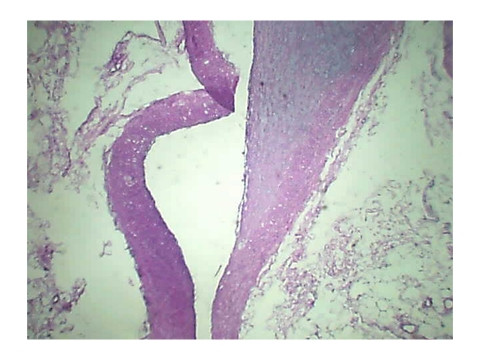
**Occasional thrombi in the blood vessels**.

**Figure 4:**
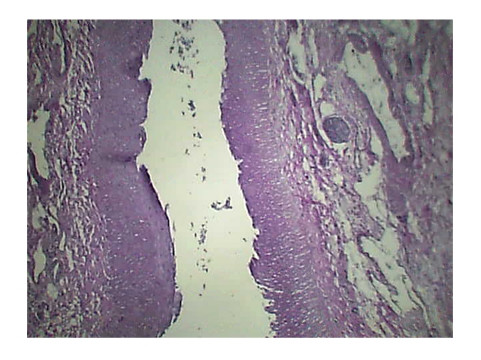
**slightly thickened blood vessels in the red pulp**.

**Figure 5:**
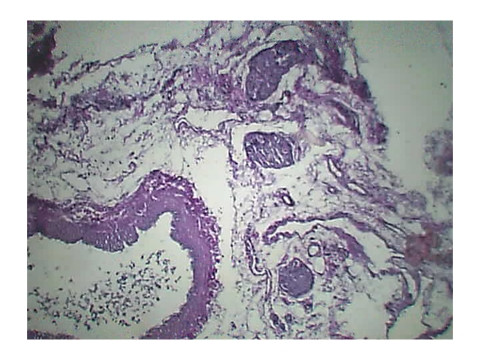
**slightly thickened blood vessels in the red pulp**.

Histopathology of Stomach revealed many variable sized AV malformations. These were present in all the layers of the stomach from the serosa to the sub mucosa and even involving the muscularis mucosa. Overlying gastric mucosa displayed reactive changes [Figure 1, Figure 2] There were occasional thrombi in the blood vessels [Figure 3]. The resected margins contained small AV malformation. The section of spleen revealed multiple AV malformation in the hilum as well as splenic trabeculae. The red pulp was markedly congested. There were slightly thickened blood vessels in the red pulp [Figure 4, Figure 5].

## Review

Upper gastro-intestinal (UGI) bleeding can be classified into several broad categories based upon anatomic and pathophysiologic factors. Peptic ulcer disease; 55 percent, Oesophagogastric varices; 14 percent, Arterial, venous, and other vascular malformations; 7 percent, Mallory-Weiss tears; 5 percent, Erosions; 4 percent, Tumors; 4 percent and other causes; 11 percent [1]. Gastrointestinal vascular diseases include angiodysplasia, arteirovenous malformation (AVM), cavernous haemangioma, hereditary haemorrhagic telangiectasia (Rendu-Osler-Weber disease), Gastric antral vascular ectasia and Dieulafoy's lesion (DL) [1,2].

Angiodysplasia presents as an irregular shaped clusters of ectatic small arteries, small veins and their capillary connections. These lesions are called by various names such as vascular ectasia or angiectasia. Arteriovenous fistulae, often called "malformations," may be congenital or acquired. AVM remains a relatively rare clinical lesion consisting of abnormal shunts between the arterial and venous vascular systems, the diagnosis of which is problematic because routine barium contrast studies and endoscopy fail to demonstrate the lesion. With increasing use of angiography over the past 30 years in the assessment of gastrointestinal bleeding, AVM has been more frequently recognized [3]. Gastric AVM may clinically be asymptomatic or may present as massive upper gastrointestinal bleeding or chronic iron deficiency anaemia [4]. Gastric antral vascular ectasia (GAVE or watermelon stomach) is a rare cause of UGI bleeding. It is often confused with portal hypertensive gastropathy, both of which can occur in patients with cirrhosis [4,5]. The term watermelon stomach is derived from the characteristic endoscopic appearance of longitudinal rows of flat, reddish stripes radiating from the pylorus into the antrum which resemble the stripes on a watermelon [1]. The red stripes represent ectatic and sacculated mucosal vessels. Dieulafoy's Lesion (DL) is an uncommon cause of gastric bleeding. It accounts for less than 5% of all gastrointestinal bleeds in adults [2]. However, unlike most other aneurysms these are thought to be developmental malformations rather than degenerative changes. DL lesion has also been given other names: caliber-persistent artery, gastric arteriosclerosis, cirsoid aneurysm, and submucosal arterial malformation. Majority of the Dieulafoy's lesions occur in the upper part of the stomach, however they can occur anywhere in the GI tract. Extragastric DLs are uncommon, but have been identified more frequently in recent years because of increased awareness of the condition. Duodenum is the commonest location (18%) followed by colon (10%) and jejunum (2%) and oesophagus (2%) [2]. The pathology of the lesion is essentially the same. The most common presenting symptom is recurrent, often massive haematemesis associated with melaena (51%). The lesion may present with haematemesis alone (28%), or melaena alone (18%) [5,6]. Clinical symptoms may include perforation or haemoperitoneum. Characteristically, there are no symptoms of dyspepsia, anorexia or abdominal pain. Initial examination may reveal haemodynamic instability, postural hypotension and anaemia. The mean hemoglobin level on admission has been reported to be between 8.4–9.2 g/dl in various studies [7,8]. The average transfusion requirement for the initial resuscitation is usually in excess of three and up to eight units of packed red blood cells [9,10]. Dieulafoy's is inherently a difficult lesion to recognize, especially when bleeding is inactive. In approximately 4–9% of massive upper gastrointestinal haemorrhage, no demonstrable cause can be found [10,11]. Dieulafoy's lesion is thought to be the cause of acute and chronic upper gastrointestinal bleeding in approximately 1–2% of these cases [12,13]. It is thought to be more common in males (M: F = 2:1) [13,14] with a median age of 54 years at presentation [14,15]. Approximately 75% to 95% of Dieulafoy's lesions are found within 6 cm of the gastroesophageal junction, predominantly on the lesser curve [16]. The blood supply to that portion of the stomach is from a large submucosal artery arising directly from the left gastric artery.

Osoephagogastroscopy (OGD) can successfully identify the lesions in approximately 82% of patients. Approximately 49% of the lesions are identified during the initial endoscopic examination, while 33% require more than one OGD for confident identification [17-19]. The remainder of the patients with Dieulafoy's lesions is identified intraoperatively or angiographically [20,21]. Endoscopic ultrasound can be a useful tool in confirming the diagnosis of a Dieulafoy's lesion, by showing a tortuous submucosal vessel adjacent to the mucosal defect. Angiography, during active bleeding has been helpful in a small number of cases in which initial endoscopy failed to show the bleeding source. It has been tentatively suggested that, in selected cases where experienced radiological, endoscopic and surgical staff are available, thrombolytic therapy to precipitate bleeding can be used electively as an adjunct to diagnostic angiography to help in localizing Dieulafoy's lesion [22]. Other reported diagnostic methods include CT and enteroclysis [23]. For acute and massive gastrointestinal haemorrhage, immediate embolisation can stop bleeding and maintain vital signs of positive bleeders [24]. Endoscopic techniques used in the treatment include epinephrine injection followed by bipolar electrocoagulation, monopolar electrocoagulation, injection sclerotherapy, heater probe, laser photocoagulation, haemoclipping or banding [2]. Rarely, surgical removal of the lesion may be needed and is recommended only if other treatment options have not been successful. Endoscopic therapy is said to be successful in achieving permanent haemostasis in 85% of cases. Of the remaining 15% in whom re-bleeding occurs, 10% can successfully be treated by repeat endoscopic therapy and 5% may ultimately require surgical intervention [19,25]. The endoscopic criteria proposed to define DL are: 1) Active arterial spurting or micropulsatile streaming from a minute mucosal defect or through normal surrounding mucosa, 2) Visualization of a protruding vessel with or without active bleeding within a minute mucosal defect or through normal surrounding mucosa, and 3) Fresh, densely adherent clot with a narrow point of attachment to a minute mucosal defect or to normal appearing mucosa [24,26]. DL is characterized by a single large tortuous arteriole in the submucosa which does not undergo normal branching, or one of the branches retain high caliber of about 1–5 mm which is more than 10 times the normal diameter of mucosal capillaries. The lesion bleeds into the gastrointestinal tract through a minute defect in the mucosa which is not a primary ulcer of the mucosa but erosion probably caused from the submucosal surface by the pulsatile arteriole protruding into the mucosa [2]. It has also been suggested that a congenital or acquired vascular malformation might be the underlying cause [25,26]. Histologically, the eroded artery appears normal. There is no evidence of any mucosal inflammatory process, signs of deep ulcerations, penetration of the muscularis propria, vasculitis, aneurysm formation, or arteriosclerosis [6,27,28]. Patients with lesions in the duodenal bulb and proximal jejunum, present in a similar way to those with gastric lesions. Patients with lesions in the middle or distal jejunum, right colon and rectum present with massive rectal bleeding [29,30]. The risk of re-bleeding after endoscopic therapy remains high (9 to 40 percent in various reports) due to the large size of the underlying artery [31,32]. The mortality rate for Dieulafoy's was much higher before the era of endoscopy, where open surgery was the only treatment option [33,34]. Hence vascular diseases of GIT are a known but rare cause of upper or lower GIT bleeds. It may present as a diagnostic challenge because of its diverse manifestations, however, a physician should always consider vascular diseases as a cause of recurrent unexplained GI bleed [35]. Management of AVM may warrant major surgical undertaking both in elective as well as in emergency situation [[4,16], and [35]].

Our patient had a diffuse type of AV malformation involving whole of the stomach as well as spleen which is an unusual occurrence. Attempt to diagnose by endoscopy lead to massive bleeding causing severe haemodynamic instability requiring emergency exploratory laparotomy and total gastrectomy with spleenectomy. AVM are more and more treated by endoscopic and endovascular techniques during the last twenty years but surgery remain a major rescue tool in emergency and treatment option in elective situations.

## Consent

Written informed consent was obtained from the patient for publication of this case report and accompanying images. A copy of the written consent is available for review by the Editor-in-Chief of this journal.

## Competing interests

The authors declare that they have no competing interests.

## Authors' contributions

MIK carried out management of the patient and prepared the manuscript. MTB carried out diagnostic procedures and also helped in drafting the manuscript. MFB helped in preparing manuscript and review of literature. NM carried out Gynaecological management of the patient and helped in drafting the manuscript.
